# The Role of Chromatin Density in Cell Population Heterogeneity during Stem Cell Differentiation

**DOI:** 10.1038/s41598-017-13731-3

**Published:** 2017-10-17

**Authors:** Mahdi Golkaram, Jiwon Jang, Stefan Hellander, Kenneth S. Kosik, Linda R. Petzold

**Affiliations:** 1 Department of Mechanical Engineering, University of California, Santa Barbara, Santa Barbara, CA USA; 2 Department of Molecular, Cellular, and Developmental Biology, Neuroscience Research Institute, University of California, Santa Barbara, Santa Barbara, CA USA; 3 Department of Computer Science, University of California, Santa Barbara, Santa Barbara, CA USA; 4 Department of Information Technology, Division of Scientific Computing, Uppsala University, SE-75105 Uppsala, Sweden

## Abstract

We incorporate three-dimensional (3D) conformation of chromosome (Hi-C) and single-cell RNA sequencing data together with discrete stochastic simulation, to explore the role of chromatin reorganization in determining gene expression heterogeneity during development. While previous research has emphasized the importance of chromatin architecture on activation and suppression of certain regulatory genes and gene networks, our study demonstrates how chromatin remodeling can dictate gene expression distribution by folding into distinct topological domains. We hypothesize that the local DNA density during differentiation accentuate transcriptional bursting due to the crowding effect of chromatin. This phenomenon yields a heterogeneous cell population, thereby increasing the potential of differentiation of the stem cells.

## Introduction

Recent advances have enabled mapping of the local structure of chromatin by analyzing the complement of DNA-associated proteins and their modifications along chromosomes. The introduction of the chromosome conformation capture (3C) methods by Dekker *et al*.^1^ opened new windows toward a better understanding of chromatin structure. Using spatially constrained ligation followed by locus-specific polymerase chain reaction (PCR), 3C provides information regarding long-range interactions between specific pairs of loci. Other researchers extended the 3C technique to develop chromosome conformation capture (3C)-on-chip (4C)^2,3^, and 3C-Carbon Copy (5C)^4^ as fast and unbiased technologies to further study the nuclear architecture. The introduction of the Hi-C method by Lieberman *et al*.^5^ facilitated obtaining the frequency of interactions between all genomic loci in a single experiment. Finally, Fullwood *et al*.^6^ presented chromatin interaction analysis by paired-end tag sequencing (ChIA-PET) for direct analysis of chromatin interactions exclusively to those formed between sites bound by a given DNA- or chromatin-interacting protein.

Recently, Hi-C data has been extensively used to explore chromatin reorganization, by primarily focusing on topologically associating domains (TAD). Such studies revealed the underlying principles of chromatin organization^5^ and its variability across single cells of the same population^7^, among distinct human cell lineages^8,9^, and between different species^10,11^. The importance of chromatin remodeling in gene regulation^12–15^ has provided compelling evidence that chromatin remodeling can activate or suppress certain genes and can control gene expression profiles, in particular during embryonic stem cell differentiation^16–18^. However, the role of chromatin in inducing heterogeneity in a cellular population has been underappreciated. Here we introduce local chromatin (DNA) density to describe how chromatin condenses differentially in the vicinity of certain gene loci and how this can influence the gene expression profile.

It is well established that phenotypic cell-to-cell variability observed in a clonal cell population is due to gene expression fluctuations^19–22^. Substantial evidence^22–26^ supports fast binding and unbinding of RNA polymerase II and transcription factors in transcriptional bursting, which contributes to such fluctuations (noise) at the population level. However, these fluctuations can serve regulatory roles^27–29^. For instance, the experimental reduction of protein expression noise in *Bacillus subtilis* ComK, the regulator for competence for DNA uptake, leads to a decrease in the number of competent cells^27^. Moreover, stochastic activation of both, either one or none of the two alleles of a gene in a heterozygote organism can contribute to the phenomenon of hybrid vigor^28^, or as demonstrated by many reports, gene expression noise can lead to differentiation of unicellular organisms into two distinct states of gene expression such as lysis-lysogeny decision in lambda phage-infected *E. coli* as well as the lac operon in *E. coli* ^28^.

Chang *et al*.^30^ showed that in clonal populations of mouse haematopoietic progenitor cells, those with higher and lower Sca-1 expression are capable of reconstituting the whole population distribution of Sca-1 expression, suggesting hidden multi-stability within one cell type. In particular, several studies have highlighted the vital function of gene expression heterogeneity in cell fate decision^31^. However, despite the convincing evidence underpinning the necessity of heterogeneity in a cell population for stem cells to differentiate, the direct mechanism that governs this heterogeneity and its association with chromatin reorganization during development has not been determined.

## Results

In^32^ we illustrated that macromolecular crowding can control gene expression fluctuations. Here we present a model based on the macromolecular crowding effects of chromatin structure to reveal the underlying principles of population heterogeneity and its regulation. By employing Hi-C interaction data^8^, we identify the DNA density as a novel element playing a vital role in modulation of gene expression heterogeneity. We verify our model predictions using several new experiments as well as recent single cell RNA-seq data^33^. Our model predicts an increase in population heterogeneity during development, an increased chromatin density of the majority of genes, and an increase in bimodal behavior. Contrary to the current paradigm of epigenetic regulation, the role of chromatin is beyond a mere on and off switch for certain genes: it contains information, even in the unoccupied space of the nucleus, that serves regulatory purposes to control population heterogeneity. In this work we mainly focus on genes with differential heterogeneity but similar mean expression.

Recent discoveries that analyze TADs and chromatin looping interactions^34^, as well as recent tools to facilitate building a three-dimensional model of chromatin, allow an unprecedented accuracy^35–39^. Here we first introduce the idea of chromatin density as a regulatory component of gene expression in a cell population. Interphase chromatin is compartmentalized into tightly packed (heterochromatin) and less compact domains (euchromatin) which are localized primarily to the peripheral and inner nucleus, respectively. Gene repression and activation are highly associated with this packaging, and controlled by histone modifications. Trimethylation at H3K4, H3K36, or H3K79 can result in an open chromatin configuration and is, therefore, characteristic of euchromatin, whereas condensed heterochromatin is enriched in trimethylation of H3K9, K3K27, and H4K20^40^. Furthermore, DNA methylation, histone modification and variants contribute strongly to gene activation and repression during early embryonic development^41^. Aside from this paradigm, the importance of local variation of the chromatin density even within the same chromatin state – heterochromatin or euchromatin – and its regulatory role have not been fully addressed.

**Figure 1:**
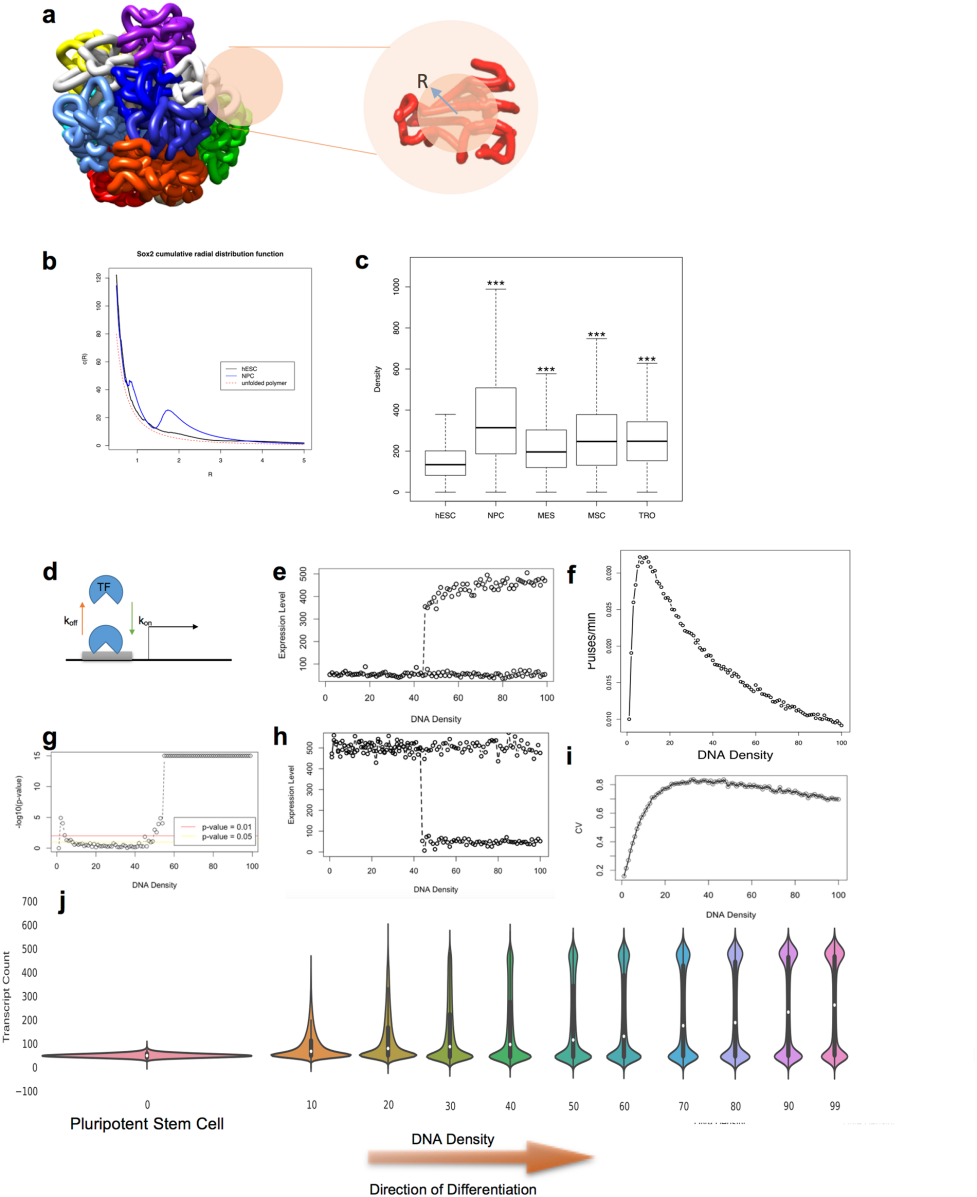
Macromolecular crowding model of gene expression. (**a**) MCMC simulations show a lack of inter-chromosomal entanglement. Schematic illustration of the density sphere around an arbitrary gene locus is provided on the right. (**b**) cumulative radial distribution function of DNA bps around the SOX2 gene locus demonstrates the local density variation between hESC and NPC. (**c**) density increases significantly during hESC differentiation (***p-value < 0.001). (**d**) binary model of gene expression for gene activating and repressing transcription factors. (**e** and **f**) bifurcation diagrams of gene expression for transcriptionally activated (**e**) and repressed (**h**) genes. (**f**) pulse frequency analysis of transcriptional bursting. (**g**) bimodality inspection during chromatin condensation by Hartigan’s Dip test. (**i**) population heterogeneity induced by transcriptional bursting and chromatin condensation. (**j**) steady state gene expression distribution variation during stem cell differentiation aroused from chromatin condensation.

We introduce Γ as a parameter for quantification of the local chromatin density (which we will henceforth simply refer to as density) as follows. For any gene X at a certain genomic coordinate j, we imagine a small sphere with a radius R centered at j, and count the number of base pairs (bps) that lie inside this sphere. To be more precise, suppose that x coincides with the transcription start site (TSS) of a gene X. Due to the relatively low resolution of the current Hi-C experiment (~40 Kbps) – which will be discussed later – the center of the density sphere (DS) will be approximated as the start or the end of the genomic coordinates of gene X, depending on whether transcription occurs on the positive or the negative strand. The second assumption in the evaluation of the density is that any inter-chromosomal interactions are neglected. This assumption is of course valid only due to the lack of entanglement and insignificant inter-chromosomal interactions, as shown by the fractal globule model^5^. This can also be verified using Markov Chain Monte Carlo (MCMC) sampling simulation results^38^ (Fig. 1a shows the lack of inter-chromosomal entanglement). Given that bps in proximity tend to show stronger interactions, it has been proposed that the physical distance of two bps is proportional to 1/IF_ij_^e^, where e is a constant^38^. IF_ij_ is the (i, j)th element of the interaction frequency matrix of the chromosome in which gene X is located as obtained by Hi-C data. Rousseau *et al*.^38^ used leave-one-out cross-validation and MCMC to show that e ~ 1–3 is the best range for e (e = 1 is used in this study for simplicity). The density Γ_j_ can then be computed as:

1 $${{\rm{\Gamma }}}_{{\rm{j}}}=\,\sum _{{\rm{i}}}{\rm{U}}(\frac{1}{{{{\rm{I}}{\rm{F}}}_{{\rm{i}}{\rm{j}}}}^{{\rm{e}}}}-{\rm{R}})$$

where U(t) is the characteristic function defined as 1 for t > 0 and 0 otherwise. One might simply discard those elements of the interaction frequency matrix with zero interaction (insignificant interactions are also often set to zero during the quality control step of the Hi-C data preparations). Note that the density Γ = d_1_ therefore corresponds to d_1_ * 40,000 base pairs in the DS. The drawback of the above definition of the density is its dependence on the radius of the density sphere R. While larger radii account for more bps around the gene of interest, they do not properly reflect the local variation in the chromatin compactness. We define the cumulative radial distribution function c(R) as Γ/V, to assess the sensitivity of Γ to R (V is the volume of the density sphere).

In this study, publicly available, normalized intra-chromosomal Hi-C data by Dixon *et al*.^8^ for five cell lines (H1 human embryonic stem cell (hESC) and H1-derived cells including neural progenitor cell (NPC), mesenchymal stem cell (MSC), mesendoderm cell (MES), and trophoblastic cells (TRO)) were used to obtain the DNA densities. Figure 1b illustrates a significant change in c(R) at the SOX2 promoter site for hESC vs NPC cell lines. It can be seen from Fig. 1b and^16^ that chromatin is condensing in the vicinity of the SOX2 gene during differentiation from hESC to NPC, which is particularly interesting because hESC and NPC show similar expression levels of SOX2^42^. A relatively decondensed chromatin state in hESC can also be inferred by comparing c(R) at the SOX2 gene locus and unfolder polymer (it can be shown that c(R) ~1/R^2^ for an unfolder polymer). We then investigated the density around all genes using R = 2 (recommended by^43^) (Fig. 1c), suggesting a significant global increase in the density among all of the four differentiated cell lines studied (p-value ≪ 0.001) (Table S1). Further analysis showed that this conclusion is insensitive to R for R ~ 1–3 (Fig. 1b shows a similar trend in the density of the SOX2 gene during NPC differentiation regardless of R i.e. the model is robust with respect to the choice of R in this range). Note that given the experimental value of 0.0123 bp/nm^3^ for the density of human nuclei, R = 1 can be scaled to a physical distance of ~250 nm (similar scale as an average TAD). One might argue that single-cell variation in chromatin interactions can affect this study and therefore, this mode should be trained using single-cell Hi-C data. However, as suggested by^7^ individual chromosomes maintain domain organization at the megabase scale (size of a DS) and cell-to-cell variations in chromosome structure occur at larger scales. We conclude that chromatin experiences a global condensation during differentiation, measured by the density parameter Γ. In addition, we should mention that by analyzing the high resolution promoter capture Hi-C data from the recently published research by Freire-Pritchett *et al*.^63^, we observed that the measured densities are significantly correlated (p-value of correlation test <2.2e-16) with our previously measured densities from 40 kb resolution Hi-C^8^, demonstrating that our conclusions are robust to the resolution of Hi-C data.

Higher levels of chromatin looping in differentiated cells is not surprising. For example, it has been shown that regions of condensed heterochromatin form during pluripotent embryonic stem cell differentiation, and silencing histone marks accumulate^16^, which result in differential expression in daughter cells. However, we also found density changes in highly expressed genes (i.e. SOX2) and in genes whose mean expression levels remain unchanged, as shown below. These results suggest that, beyond our understanding of the role of the chromatin reorganization limited to gene silencing and activation, chromatin conformation might be involved in other types of gene regulation such as gene expression heterogeneity.

The cellular environment is packed with DNA, RNA, proteins and other macromolecules hindering the diffusion of other molecules such as transcription factors and RNA polymerases. Several studies^32,44,45^ have demonstrated the impact of macromolecular crowding on the rebinding time of DNA binding proteins (DBP), leading to heterogeneity in gene expression. In particular, *in vitro* experimental studies by Muramatsu *et al*.^46^ show that macromolecular crowding can alter the diffusion and the biochemical rates of globular proteins in concentrated protein solutions. It has been suggested by Morelli *et al*.^47^ that the effect of crowding can be taken into account by merely scaling the association (k_on_) and dissociation rate constants (k_off_) of DBPs. Thus,

2 $$\frac{{k}_{on}}{{k}_{on}^{0}}={{\rm{\Gamma }}}^{-\gamma }\,{\rm{and}}\,\frac{{k}_{off}}{{k}_{off}^{0}}={{\rm{\Gamma }}}^{-\gamma -1},$$

where k_on_^0^ and k_off_^0^ are the basal association and dissociation constants of DBP and where γ ≈ 0.36 (Table S2). Note that k_on_^0^ and k_off_^0^ are chosen such that the obtained k_on_ and k_off_ using equation (2) for a gene with an average local DNA density lies within the range of the reported experimental data for association and dissociation rate constants of DNA binding protein (Table S2). Equal k_on_^0^ and k_off_^0^ are considered for all genes which can be explained due to the diffusion-limited kinetics of DBPs. In other words, while association and dissociation rates of DBPs to DNA can vary due to factors such as promoter sequence, DNA methylation pattern, etc., the influence of these factors is negligible compared to the effect of the variation the diffusion rates of DBPs which is explained by parameter Γ. The following major assumptions have been made in our macromolecular crowding (MMC) model: (1) *macromolecular crowding is directly correlated to the DNA density*. DNAs are wrapped around histone complexes and are bound by many proteins such as RNA polymerases, transcription factors, mediators, and cofactors. Therefore, we assume that the DNA density is correlated with local molecular crowding. Hence, we assume that high density regions of chromatin are enriched in other proteins, histones and other molecules. The DNA density alone, therefore, can simply represent the concentrations of other bound molecules as well. (2) *RNA polymerases and/or TFs will only diffuse locally during any rebinding events*. In other words, we assume that the diffusive particles are less likely to exit the density sphere with radius R, due to the highly packed chromatin structure which restricts the diffusive particle to the vicinity of the promoter^43^. (3) *basal association and dissociation biochemical rates do not vary significantly among different genes*. (4) *1D sliding of TF on DNA is ignored*. The sliding length of TF on DNA has been measured to be ~30–900 bps^48^. Given the current resolution of Hi-C data (40 Kbps), precise analysis of the impact of the density alteration on 1D sliding of TF on a DNA strand is not feasible.

We utilized the described simple model to study the influence of the chromatin density on the gene expression patterns by simulating a two state (binary) system (Fig. 1d). The Gillespie stochastic simulation algorithm (SSA)^49^ was used (Materials and Methods) to simulate the constructed biochemical system (note that here we assume a time-homogeneous Markov process in order to use SSA). We then inspected the transcriptional bursting behavior for different DNA densities by exploring the pulsing frequency, the coefficient of variation (CV) of RNA distributions, and the bimodality of gene expression in a cell population (Fig. 1e–j). Our simulation results show a general decrease in pulse frequency, suggesting slower expression kinetics, which could be expected due to the limited diffusion of TF (or RNA polymerase) by crowding. Figure 1j depicts steady state RNA distribution variation caused by the increase in DNA density. At very low densities, the MMC model predicts a unimodal normally distributed cell population with a relatively low variation induced by gene intrinsic noise. During stem cell differentiation, chromatin condenses and the DNA density around several promoters increases. Chromatin condensation is followed by transcriptional bursting and heterogeneity in a cell population generating a long tail RNA distribution with high variation. Ultimately, a second cell state emerges due to high RNA variation (measured by CV), resulting in a bimodal RNA distribution. Further increase in the DNA density gradually eliminates the intermediate states^32^, indicated by a decrease in the CV (Fig. 1i). While Fig. 1i initially illustrates an increase in the CV, upon emergence of the second cell state (determined by Hartigan’s Dip test for unimodality^50^), the CV tends to decrease. Our simulation results suggest an analogous trait whether the gene is transcriptionally turned on or off (Fig. 1e and h). While both undergo a bifurcation, repressive TFs endow a second state with a lower expression level. The bifurcation predicted by our model is consistent with the previous reports on several developmental genes, namely, GATA-1 and PU.1 in a myeloid progenitor cell^51^. All in all, it can be inferred from the MMC model that chromatin reorganization can alter the excluded volume around certain gene loci and thereby can modify the rebinding kinetics of TFs, leading to distinct gene expression patterns. This explains how unoccupied space of the nucleus encodes vital information regarding gene expression heterogeneity. While only one state is accessible at lower densities, increasing the densities can increase the accessibility of other states by rendering wider RNA distributions. Further increase in the densities imposes an all or nothing response which entails a bifurcation toward a second stable state with lower or higher expression levels depending on whether the gene is transcriptionally activated or repressed.

Based on our simulation results, we hypothesize the following:

1. During differentiation the macromolecules accumulate around the promoters of different genes, intensifying the effect of macromolecular crowding.
2. An increase in the chromatin density increases transcriptional bursting due to macromolecular crowding.
3. Transcriptional bursting increases the CV of RNA expression in a cell population. Hence, the CV increases during differentiation and several genes undergo a bifurcation stochastically, allowing a portion of cells to enter a new cell state while the remainder retain their current cell state.
4. This process creates the potential for pluripotent cells to differentiate.

To test how density changes are related to gene expression variation during stem cell differentiation, we focused on NPC differentiation. We used scRNA-seq data by Chu *et al*.^33^ to assess gene expression variation in H1 hESC and H1-derived NPC because they used the same dual SMAD inhibition method^52^ to differentiate hESC to NPC with Dixon *et al*., which enables direct comparison between DNA density and gene expression variation. First, raw scRNA-seq data of H1 hESC (212 cells) and NPC (173 cells) were counts per million (CPM) normalized. A significant dependency of the CV of RNA expression distributions on their mean expression can be seen, which is due to inherent high technical noise of scRNA-seq experiments (Fig. S1a and b). To account for any bias, we first discretized the expression levels of all genes within each cell line into 50 logarithmically-spaced intervals, assuming that the mean expression level of those genes that lie inside each interval is similar. For each interval, the biological variation of the genes with low CV is strongly confounded by their technical variation. Therefore, we exclude any genes with CV less than 3 standard deviations (CV < 3stdev), generating a list of genes with high biological variation. Note that since the technical variations of the genes with similar mean expression levels can be assumed to be similar, the obtained list of genes includes only those genes whose biological variations are significant compared to their technical noise. Finally, to account for the technical variation among cell lines, we analyzed only genes whose mean expression is not statistically different (q-value > 0.01) between two cell lines (hESC and NPC).

**Figure 2:**
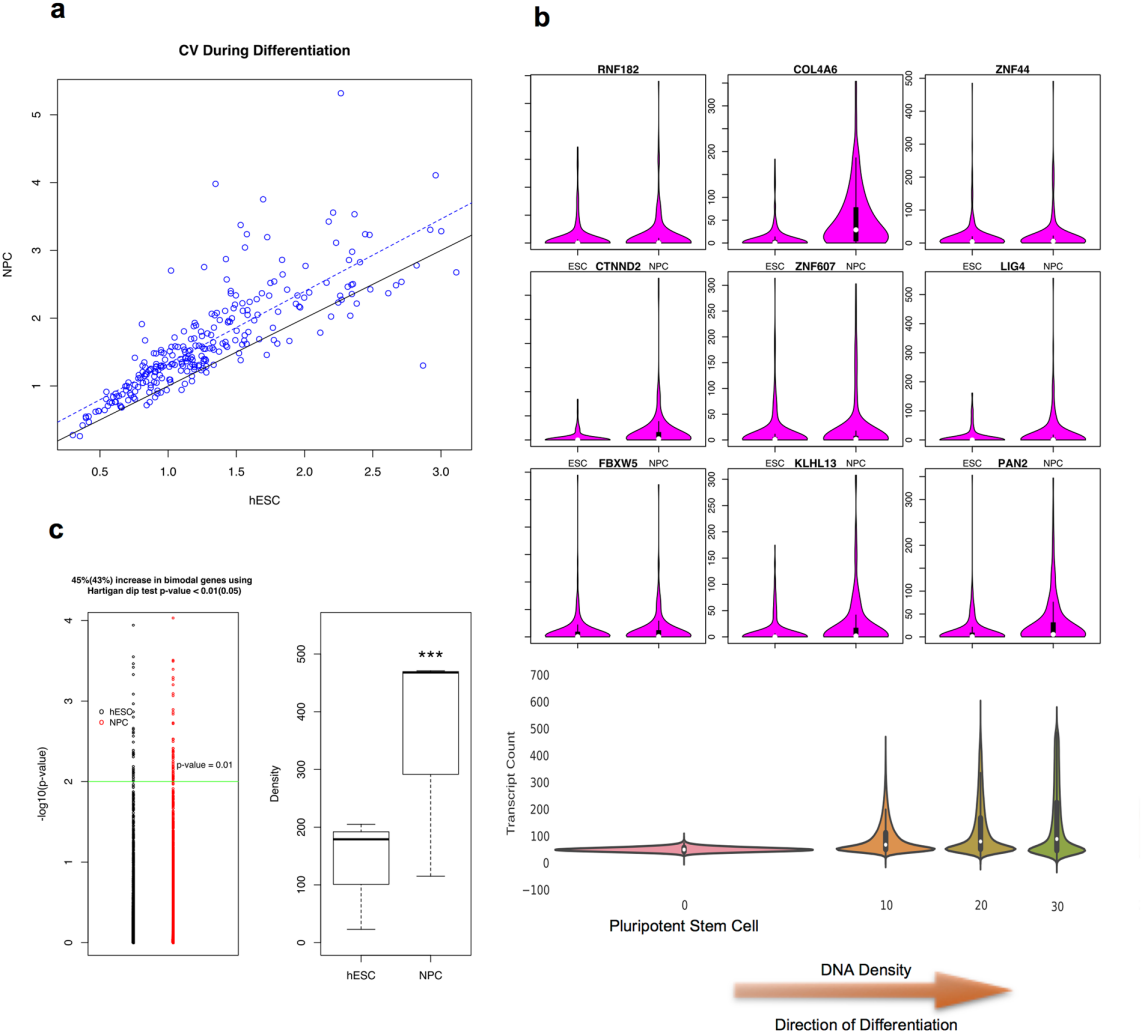
Analysis of gene expression heterogeneity during development. (**a**) CV increases during NPC differentiation for the majority of genes. (**b**) long tailed steady state gene expression distributions appear as a result of chromatin condensation, as predicted by the MMC model. (**c**) unimodal to bimodal switch during NPC differentiation due to significant density increase (***p-value < 0.001).

Figure 2a demonstrates that the majority of the genes are more heterogeneous during differentiation, resulting in higher CV in NPC compared to hESC, as predicted by our model. This is not due to non-NPC contamination because Chu *et al*. sorted out SOX2-positive NPCs for scRNA-seq.^33^. Note that all of the genes remaining in the obtained gene list have a unimodal RNA distribution, thus the model predicts a steady state long tail unimodal RNA distribution with increasing variation from hESC to NPC. This is illustrated in Fig. 2b for 9 sample genes with high gene expression variations.

Another prediction of the MMC model of gene expression is an increased number of genes with a bimodal distribution. We argue that although as previously discussed, the CV values are confounded by the technical noise, RNA expression bimodality is less likely to be affected by that. Thus to study the bimodality we dropped out the CV > 3stdev criterion for the gene list selection and compared the number of genes which are bimodal in each cell line (p-value of Hartigan’s Dip test for unimodality <0.01). Interestingly, as predicted by our model, not only do all the bimodal genes in hESC remain bimodal in NPC, but also a 45% increase in the number of bimodal genes was observed. We then analyzed the density values of the genes that undergo a bimodal distribution switch during NPC differentiation. Again, as predicted by our MMC model, there is a significant increase in the density of the genes of interest (Fig. 2c).

**Figure 3:**
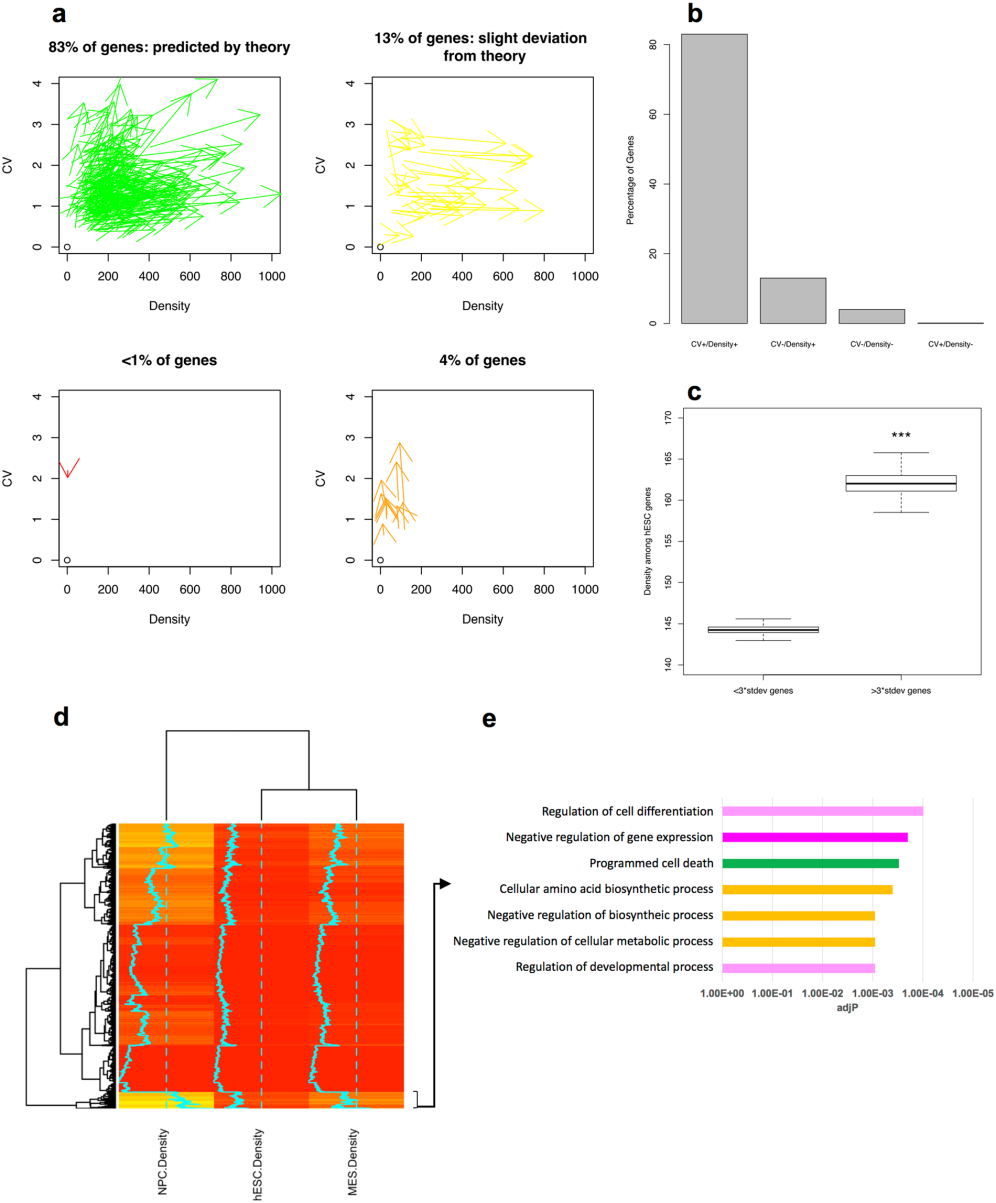
Direction of differentiation. (**a**) representation of differentiation arrows in CV-Density coordinates. (**b**) the majority of genes experience increases in both density and CV during NPC differentiation. (**c**) genes with the highest density exhibit the highest CV in hESC (quartiles are estimated using bootstrapping, ***p-value < 0.001). (**d**) hierarchical clustering shows a functional role for chromatin condensation. (**e**) gene ontology explains the role of chromatin condensation and the gene expression heterogeneity in development, metabolism, and cell death.

We introduce density-CV coordinates to facilitate an efficient assessment of the model predictions. A vital prediction of the MMC model is the direction of differentiation in this coordinate plane (Fig. 3a). Each arrow represents one gene pointing from hESC to NPC (toward differentiation). The validity of the model can be tested by incorporating Hi-C data (to obtain the densities) and scRNA-seq data (to obtain the CVs). As predicted by our model, 83% of the genes experience an increase in both the density and CV during NPC differentiation. 13% of the genes show an increase in their density while their CVs are slightly reduced (Fig. 3b). This 13% discrepancy can be due to the assumptions in the MMC model and ignoring post transcriptional modifications, cell cycle dependent genes, etc. Another explanation for the decrease in the CV of 13% of the genes is the emergence of the bimodal genes during chromatin condensation which corresponds to the second regime in Fig. 1i (a negative correlation between CV and the density is expected). However, since the analysis of scRNA-seq data shows that only a small fraction of genes illustrates a bimodal behavior, we will focus on the 83% of the genes where the CVs and densities are positively correlated. Also it is critical to note that our model doesn’t exclude several other factors that can also affect the CVs but instead emphasizes the critical role of the local DNA density on gene expression variation. This observation implies that chromatin condensation is a driving force to render a heterogeneous cell population and thus exciting other silenced cell states. One might argue that this general principle could be employed to study the genes within even one cell type. We tested this hypothesis in hESC by comparing the densities of the genes whose CVs are greater than 3 stdev of all CVs within the corresponding interval (as defined previously) with the rest of the genes. This approach enables us to minimize the impact of the technical noise and gene networks on our conclusions. As predicted by the MMC model (Fig. 3c) the set of all genes with high CV in each interval manifest significantly higher densities compared to low CV genes (p-value < 0.001). These results are also confirmed in NPC (Fig. S1c). In addition, we analyzed the recently published Assay for Transposase-Accessible Chromatin with high throughput sequencing (ATAC-seq) data by Liu *et al*.^64^ to compare the chromatin accessibility of low versus high CV genes in hESCs and did not see a major difference. These results suggest that chromatin accessibility is not related to gene expression variation. We can conclude that one function of chromatin remodeling proteins is to locally condense chromatin in the vicinity of the genes destined for heterogeneous expression.

Moreover, we perform hierarchical clustering with genes whose DNA densities change during stem cell differentiation. DE genes were excluded across hESC, NPC, and MES using publically available population RNA-seq data by Jang *et al*.^42^. The first gene cluster (444 genes) (Fig. 3d) depicts the highest modification in the densities during stem cell differentiation. Using gene ontology analysis, we found that this gene cluster is significantly associated with cell differentiation, metabolism and cell death (Fig. 3e). Namely, several key transcription factors (PRDM16, HES3, HES5, ID3) and epigenetic modifiers (KDM1A) are related to cell differentiation. The mammalian target of rapamycin (mTOR), a master regulator of cellular metabolism is in the term “Negative regulation of cellular metabolic process” together with other important metabolic genes (ENO1, CDA, PEX14). Finally, programmed cell-death related genes include PINK1, CAS9, and MUL1. These results suggest that density changes during stem cell differentiation potentially affect expression variation of genes that are involved in key biological processes.

**Figure 4:**
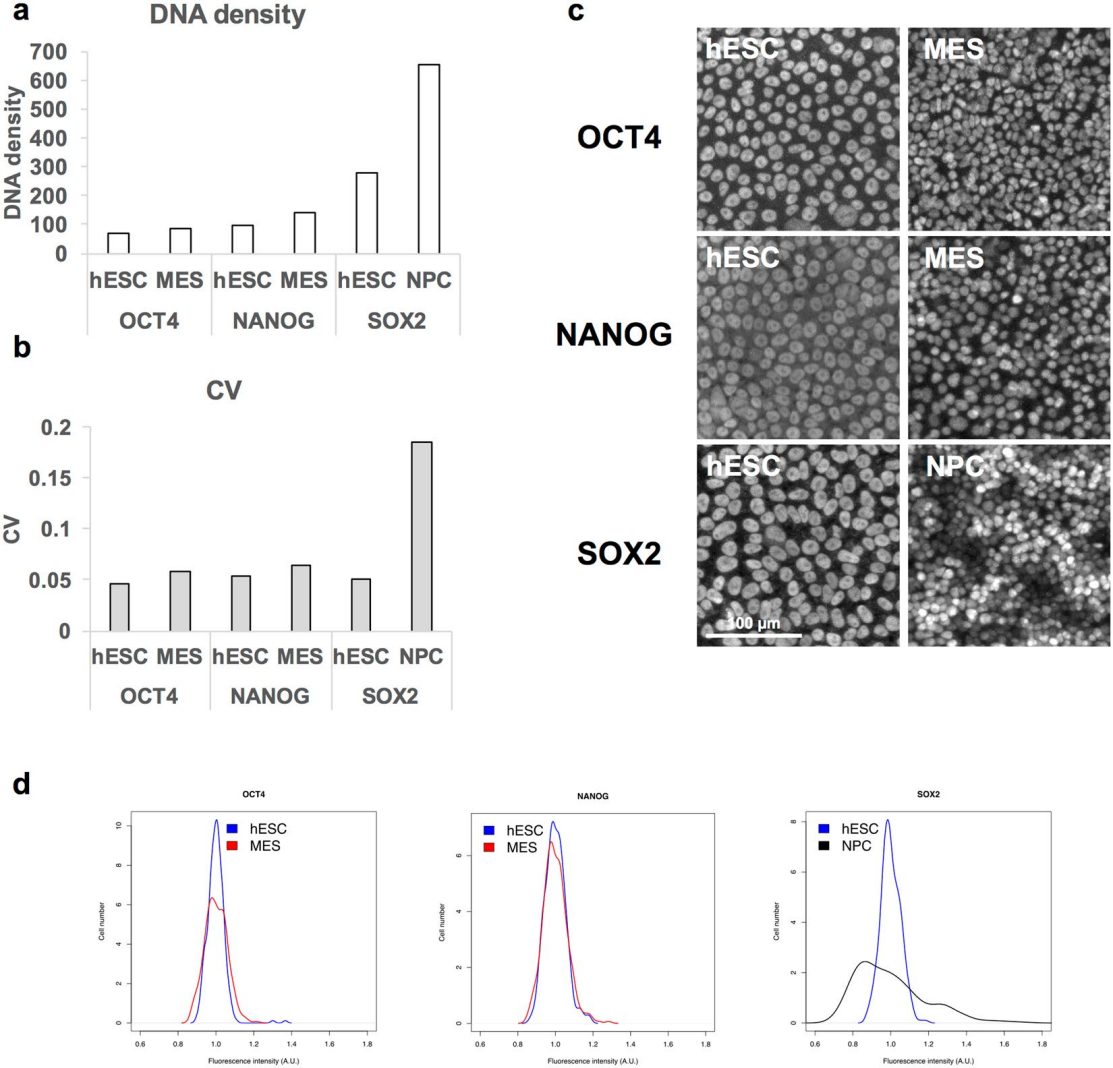
Relationship between density changes and gene expression variation in core pluripotency genes. (**a**) The densities of OCT4, NANOG, and SOX2 change during stem cell differentiation. (**b**–**d**) hESC was differentiated to NPC and MES, followed by immunostaining of OCT4, NANOG, and SOX2. CV (**b**) and gene expression distribution (**d**) were calculated from fluorescence intensity measured from individual cells (n = 315 for OCT4 in hESC, n = 351 for OCT4 in MES, n = 294 for NANOG in hESC, n = 365 for NANOG in MES, n = 308 for SOX2 in hESC, and n = 343 for SOX2 in NPC). Representative images are shown in (**c**).

OCT4, SOX2, and NANOG are core pluripotency genes that play key roles in early stem cell differentiation^53,54^. OCT4 and NANOG strongly repress NPC differentiation, while promoting differentiation into MES cells. In stark contrast, SOX2 is necessary for a NPC fate, but suppresses MES differentiation. This is consistent with the expression of these genes during stem cell differentiation, with their roles with high OCT4 and NANOG expression in MES and high SOX2 expression in NPC^42^. Given the importance of these genes for stem cell differentiation, we studied how density changes affect single cell expression patterns during NPC or MES differentiation. We limited our analysis to OCT4 and NANOG in MES differentiation and SOX2 in NPC differentiation, to exclude the potential impact of dramatic changes in mean expression levels on gene expression variation. There were no significant changes in the densities of OCT4 and NANOG during MES differentiation (Fig. 4a). Consistent with our model, when single cell expression patterns were analyzed by immunostaining, there were no substantial changes in CV and gene expression distributions (Figs 4b–d, S2). In contrast, the density of SOX2 underwent a dramatic increase in NPC compared to hESC (Fig. 4a). This change was consistent with increased CV and broadened distribution of gene expression during NPC differentiation (Fig. 4b–d). NPC derivation is also confirmed by PAX6 expression, suggesting that heterogeneous expression of SOX2 is not due to inefficient NPC differentiation (Fig. S2b). These results suggest that differentiation-induced local chromatin condensation controls heterogeneous expression of core pluripotency genes.

## Discussion

We have provided substantial evidence that population heterogeneity can be modulated by altering the local chromatin density in single cells. The current model of epigenetics suggests that gene activation and repression occur via modifications of histone H3 methylation and acetylation. Our study elucidates the functional role of higher order chromatin looping, which is regulated by CCCTC-binding factor (CTCF). Our MMC model suggests that aside from the orthodox view of loop formation in down regulation (or up regulation) of the developmental genes such as OCT4 and NANOG in NPC differentiation^55^, chromatin looping can modulate expression heterogeneity of other developmental genes (e.g. SOX2 in NPC differentiation) by adjusting the genome-wide long range interactions as well. One particular function of chromatin looping is its impact on the neighboring genes. For example, the expression variation of certain genes can be influenced by inactivation of a neighboring gene, resulting in a new, more crowded environment. It is important to note that our results show that the density not necessarily alters gene expression heterogeneity by changing the mean expression level, but that, increased densities can increase the gene expression heterogeneity even at similar expression levels.

It is also noteworthy that the current approach to study the chromatin organization by probing TADs is incapable of revealing the vast amount of information hidden in the unoccupied space of the chromatin. As illustrated by our study, chromatin reorganization can alter rebinding kinetics of TFs, through which the gene expression pattern can be modified. Recent studies^56–58^ suggest that TADs are highly conserved, not only among different cells of the same clonal population, but also across different cell types. Our study, on the other hand, expresses the local DNA density deviations among distinct cell types, specifically across progenitor and embryonic stem cells, suggesting that the local DNA density changes might play more dynamic roles than TAD reorganization during development.

## Materials and Methods

### hESC culture and differentiation

H9 hESCs (WiCell) were maintained in mTeSR1 medium (Stem Cell Technologies) on Matrigel (BD Biosciences)-coated tissue culture plates. H9 cells were passaged every 5 days by ReLeSR (Stem Cell Technologies). For NPC differentiation, hESCs were passaged as single cells using Accutase (Life Technologies) on Matrigel with ROCK inhibitor (Y-27632, Millipore) and induced to differentiate with SB431542 (10 µM, Tocris Bioscience) and NOGGIN (200 ng/ml, PeproTech)^52^. To derive MES, hESCs were plated on Matrigel-coated plates as single cells and induced to differentiate with mTeSR1 containing 5 ng/ml hBMP4 (R&D)^59^.

### Immunofluorescence

Samples were fixed with 4% paraformaldehyde for 15 min, followed by permeabilization with 0.25% Triton X-100. After blocking with 10%FBS in PBS, cells were immunostained with primary antibodies for OCT4 (Santa Cruz), NANOG (R&D), and SOX2 (Millipore) overnight at 4 °C. Staining with secondary antibodies, Alexa Fluor 555-donkey anti-mouse IgG, Alexa Fluor 555-donkey anti-rabbit IgG, Alexa Fluor 488-donkey anti-goat IgG, was done for 1 h at room temperature. Images were taken using Olympus IX71 fluorescence microscope.

### RNA isolation and quantitative PCR

Total RNA was extracted using TRIzol (Life Technologies), followed by cDNA conversion with the SuperScript III First-Strand Synthesis System (Life Technologies). Quantitative real-time PCR was performed using a QuantStudio 12 K Real-Time PCR System (Life Technologies) with Power SYBR Green PCR Master Mix (Applied Biosystems). GAPDH was used as a normalization control.

### Data acquisition and analysis

Publically available scRNA-seq data for H1 hESC and NPC cell lines were obtained from GEO using accession number GSE75748. In this study expected counts of 212 hESC cells and 173 cells NPC have been used to investigate gene expression variation^33^.

Processed FPKM-normalized RNA-seq data from H9 hESC, NPC, and MES (three replicates per cell line)^42^ were acquired from GEO using accession number GSE69982.

Hi-C intra-chromosomal interaction frequencies for 5 cell lines (H1 hESC, NPC, MES, MSC, and TRO) have been obtained used using accession number GSE52457, mapped to human genome (hg19) and normalized as described by Dixon *et al*.^8^.

All of the computations were performed using IPython 5.0, R version 3.2.2 and the BioConductor (3.3), qvalue (3.2.3), limma (3.2.4), diptest (3.2.0), GenomicRanges (3.2.3), edgeR (3.2.0), and genefilter (3.2.3). All of the figures were made using basic R graphics and packages gplots (3.2.4), and vioplot (3.2.0). All scripts used here are available from the authors upon request.

Gene ontology analysis has been performed using the web-based gene set analysis toolkit WebGestalt^60^.

### Details of simulations and computational modeling

In our study, gene expression is described using the following set of biochemical reactions:

R1 $${\boldsymbol{TF}}+{\boldsymbol{Promote}}{{\boldsymbol{r}}}_{{\boldsymbol{Repressed}}}\begin{array}{c}{{\boldsymbol{k}}}_{{\boldsymbol{on}}},\,{{\boldsymbol{k}}}_{{\boldsymbol{off}}}\\ \rightleftharpoons \end{array}{\boldsymbol{Promote}}{{\boldsymbol{r}}}_{{\boldsymbol{Active}}}\mathop{\to }\limits^{{{\boldsymbol{s}}}_{{\boldsymbol{A}}}}{\boldsymbol{M}}+{\boldsymbol{Promote}}{{\boldsymbol{r}}}_{{\boldsymbol{Active}}}$$

R2 $${\boldsymbol{Promote}}{{\boldsymbol{r}}}_{{\boldsymbol{Repressed}}}\mathop{\to }\limits^{{{\boldsymbol{s}}}_{{\boldsymbol{R}}}}{\boldsymbol{M}}+{\boldsymbol{Promote}}{{\boldsymbol{r}}}_{{\boldsymbol{Repressed}}}$$

R3 $${\boldsymbol{M}}\mathop{\to }\limits^{{{\boldsymbol{\delta }}}_{{\boldsymbol{M}}}}\varnothing $$

where k_on_ and k_off_ can be found as

$$\frac{{{\boldsymbol{k}}}_{{\boldsymbol{on}}}}{{{\boldsymbol{k}}}_{{\boldsymbol{on}}}^{{\bf{0}}}}={{\boldsymbol{\Gamma }}}^{{\boldsymbol{-}}{\boldsymbol{\gamma }}}\,{\bf{and}}\,\frac{{{\boldsymbol{k}}}_{{\boldsymbol{off}}}}{{{\boldsymbol{k}}}_{{\boldsymbol{off}}}^{{\bf{0}}}}={{\boldsymbol{\Gamma }}}^{{\boldsymbol{-}}{\boldsymbol{\gamma }}{\boldsymbol{-}}1}$$

The simulation parameters and the initial conditions are provided in the Supplementary Data Table 2. The stochastic simulation algorithm (SSA) was used to simulate the above biochemical system using the implementation in GillesPy^61^ on the MOLNS software platform^62^. For each Γ (0 < Γ < 100), 1,000 trajectories are simulated each for 1,000 minutes.

## Electronic supplementary material

Supplementary Materials

Table S1

Table S2

Table S3
