# Supplementary material for: The Role of Chromatin Density in Cell Population Heterogeneity during Stem Cell Differentiation
Source: Sci Rep. 2017 Oct 17;7:13307. doi: 10.1038/s41598-017-13731-3 (PMC5645312; doi:10.1038/s41598-017-13731-3)
Supplement: Supplementary file 1 — Supplementary Materials [file 41598_2017_13731_MOESM1_ESM.pdf]

Supplementary Materials for

The Role of Chromatin Density in Cell Population Heterogeneity during Stem Cell Differentiation

Mahdi Golkaram,* Jiwon Jang, Stefan Hellander, Kenneth S. Kosik, Linda R. Petzold

*correspondence to: m_golkaram@umail.ucsb.edu

This PDF file includes:

Figs. S1 and S2
Legends for Tables S1 to S3

Other Supplementary Material for this manuscript includes the following:

Tables S1 to S3 as Excel files

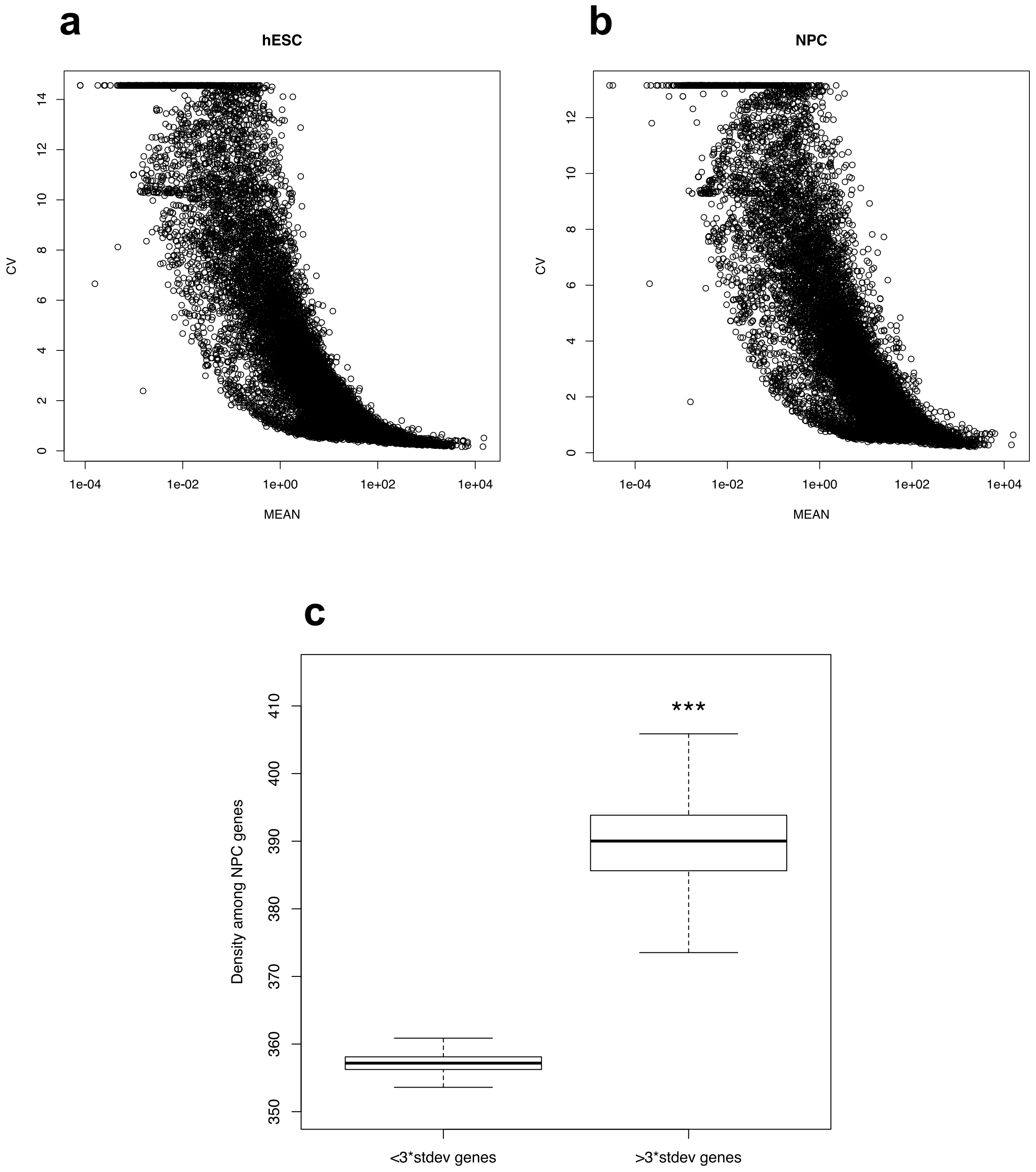

Figure S1. Analysis of scRNA-seq variations due to mean expression and DNA density variations for hESC and NPC. (**a**, **b**) scRNA-seq data show that CV strongly depends on mean expression due to technical noise in both hESC and NPC. (**c**) genes with the highest density exhibit the highest CV in NPC obtained (quartiles are estimated using bootstrapping, *** p-value < 0.001).

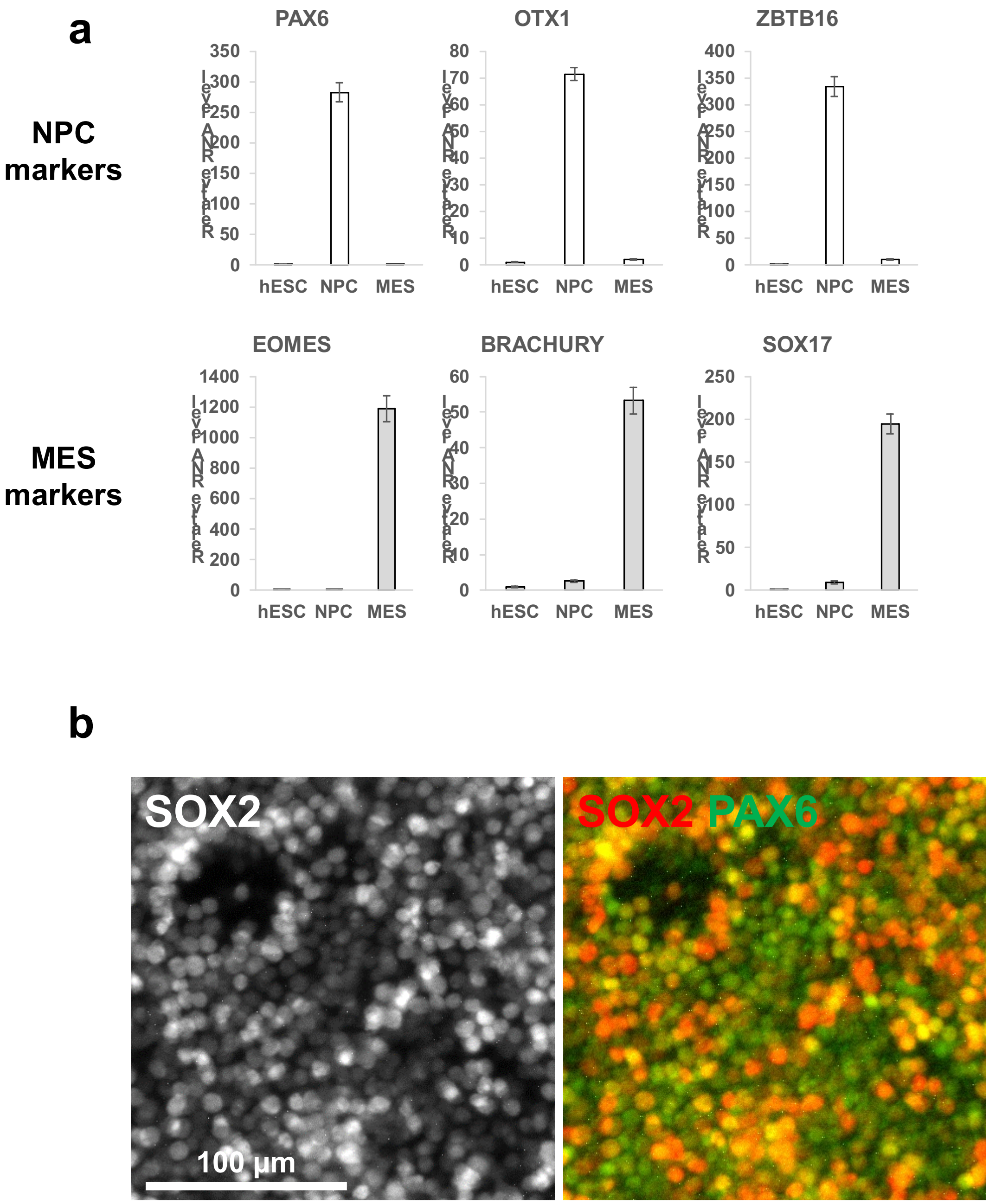

Figure S2. Validation of lineage-specific differentiation. (**a**) the expression of NPC (PAX6, OTX1, ZBTB16) and MES (EOMES, BRACHURY, SOX17) markers confirms highly specific differentiation of hESC to each lineage (n=4). Error bars represent SD. (**b**) NPC was immunostained for SOX2 and PAX6.

Table S1
Genome-wide density profiling of hESC and NPC.

Table S2
MMC simulation parameters.

Table S3
Single cell fluorescence intensity of immunostaining of OCT4, NANOG, and SOX2 normalized by mean fluorescence intensity.
